# Author Correction: Transcription tuned by S-nitrosylation underlies a mechanism for *Staphylococcus aureus* to circumvent vancomycin killing

**DOI:** 10.1038/s41467-023-39231-9

**Published:** 2023-06-23

**Authors:** Xueqin Shu, Yingying Shi, Yi Huang, Dan Yu, Baolin Sun

**Affiliations:** 1 Department of Oncology, The First Affiliated Hospital of USTC, Division of Life Sciences and Medicine, University of Science and Technology of China, Hefei, Anhui China; 2 Laboratory of Dermatology, Beijing Pediatric Research Institute, Beijing Children’s Hospital, Capital Medical University, Key Laboratory of Major Diseases in Children, Ministry of Education, National Center for Children’s Health, Beijing, China; 3 CAS Laboratory of Innate Immunity and Chronic Disease, University of Science and Technology of China, Hefei, China; 4 Hefei National Laboratory for Physical Sciences at Microscale, Hefei, China

**Keywords:** Antimicrobial resistance, Nitrosylation, Bacterial infection

Correction to: *Nature Communications* 10.1038/s41467-023-37949-0, published online 21 April 2023

In the original version of this Article, several affiliations were incorrect:

The affiliation for Xueqin Shu, Yingying Shi and Yi Huang should have been 1. Department of Oncology, The First Affiliated Hospital of USTC, Division of Life Sciences and Medicine, University of Science and Technology of China, Hefei, Anhui, China.

Baolin Sun’s listed affiliations should be: 1. Department of Oncology, The First Affiliated Hospital of USTC, Division of Life Sciences and Medicine, University of Science and Technology of China, Hefei, Anhui, China; 3. CAS Laboratory of Innate Immunity and Chronic Disease, University of Science and Technology of China, Hefei, China and 4. Hefei National Laboratory for Physical Sciences at Microscale, Hefei, China.

This has now been corrected in both the PDF and HTML versions of the Article

