# Supplementary material for: Transcription tuned by S-nitrosylation underlies a mechanism for Staphylococcus aureus to circumvent vancomycin killing
Source: Nat Commun. 2023 Apr 21;14:2318. doi: 10.1038/s41467-023-37949-0 (PMC10120478; doi:10.1038/s41467-023-37949-0)
Supplement: Supplementary file 1 — Supplementary Information [file 41467_2023_37949_MOESM1_ESM.pdf]

Supplementary information
Supplementary Figure 1

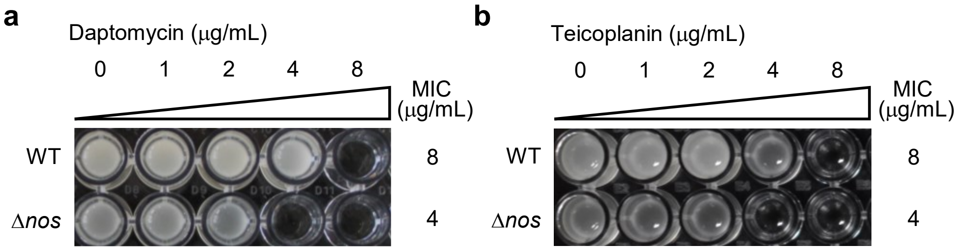

Supplementary Figure 1. The Δ*nos* mutant strain showed increased daptomycin and teicoplanin susceptibility.

a-b, MICs were assessed after the WT *S. aureus* XN108 and Δ*nos* mutant strains were grown under daptomycin (**a**) or teicoplanin (**b**) conditions in a 96-well plate with shaking at 200 rpm for 48 h at 37 ℃. Data are representative of n = 3 biological replicates.

Supplementary Figure 2

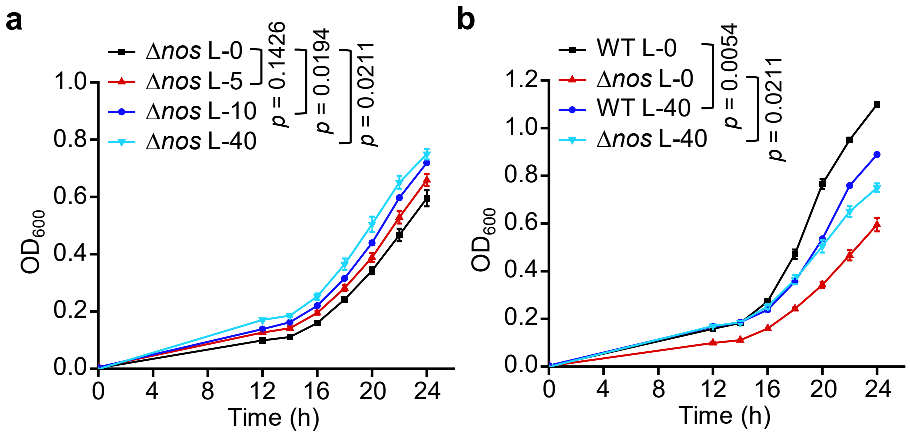

Supplementary Figure 2. The effect of NOS inhibitor L-NAME on growth.

a, The growth curve of the Δ*nos* mutant strain in MH medium containing 0, 5, 10 or 40 mM L-NAME (displayed as L-0, L-5, L-10 or L-40 respectively) in 96-well plates with an initial OD_{600} of 0.001. **b**, The growth curve of the WT and Δ*nos* mutant strains in MH medium containing 0 or 40 mM L-NAME (displayed as L-0 or L-40 respectively) in 96-well plates with an initial OD_{600} of 0.001. These cultures were incubated at 37 °C without shaking overnight (13h) and then grown with shaking at 200 rpm for 12 h. Data are means of n = 3 biological replicates with SD. Two-sided two-way ANOVA, $*p \leqslant 0.05$; $**p \leqslant 0.01$; ns, not significant. Source data are provided as a Source Data file.

Supplementary Figure 3

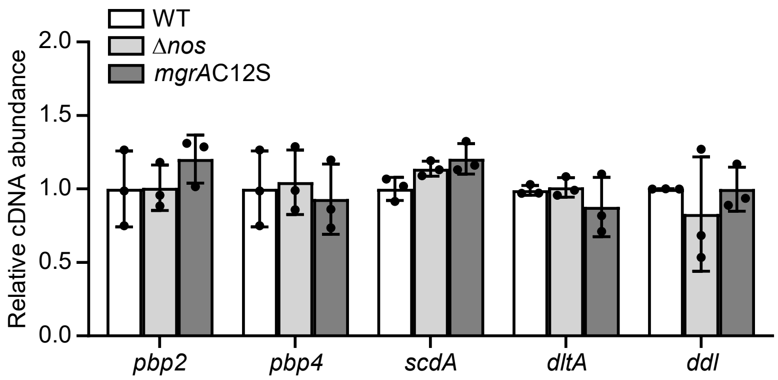

Supplementary Figure 3. The transcription level of genes involved in cell wall synthesis.

The WT, Δ*nos* or *mgrA*C12S mutant strain was cultured to mid-exponential phase (OD_{600} = 1). The transcription levels of indicated genes that were reported to regulate cell wall synthesis were assessed in all the strains by using qRT-PCR. Data are means of n = 3 biological replicates with SD. Statistical significance was calculated using the two-sided unpaired Student's t-test. The expression of these genes showed no significant difference in the Δ*nos* or *mgrA*C12S mutant strain in comparison to the WT strain. Source data are provided as a Source Data file.

Supplementary Figure 4

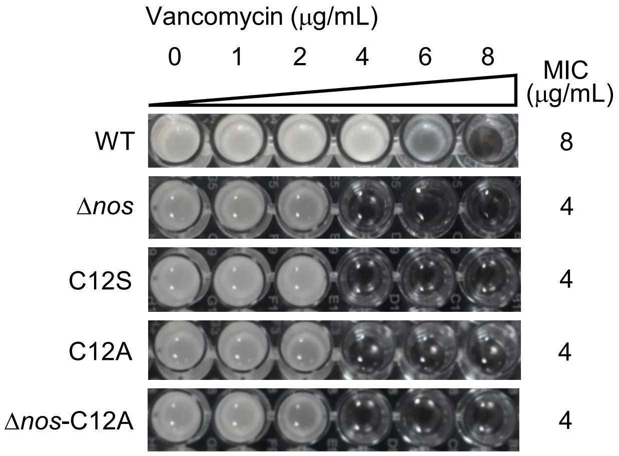

Supplementary Figure 4. The *mgrA*C12A mutant strains showed increased vancomycin susceptibility.

MICs were assessed after the WT *S. aureus* XN108, Δ*nos* mutant, *mgrA*C12S mutant, *mgrA*C12A mutant and *mgrA*C12A mutant in the Δ*nos* background were grown under vancomycin conditions in a 96-well plate with shaking at 200 rpm for 48 h at 37 °C. Data are representative of n = 3 biological replicates.

Supplementary Figure 5

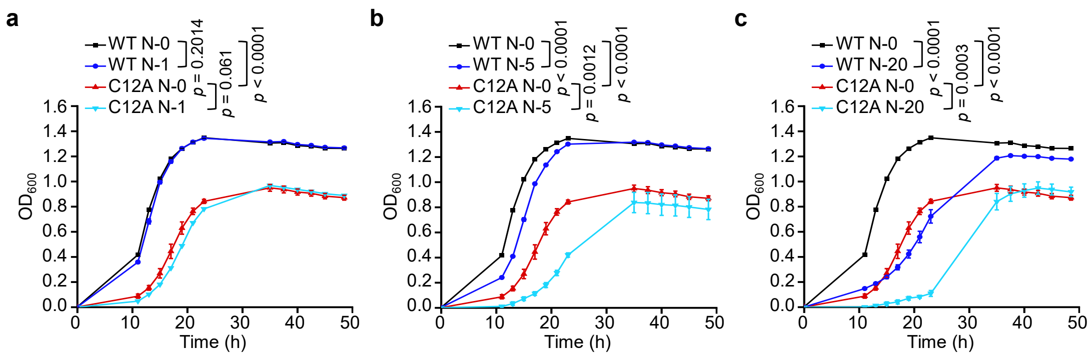

Supplementary Figure 5. The effect of NO donor SNP on the growth of the *mgrA*C12A mutant strain.

a-c, The WT and *mgrA*C12A strains were grown in MH medium containing 1 μg/mL vancomycin in 96-well plates with shaking at 200 rpm for 48 h at 37 °C. OD_{600} was monitored to evaluate the growth when different concentrations of SNP were added. N-0, N-1, N-5 or N-20 represents 0, 1, 5 or 20 μM SNP respectively. Data are means of n = 3 biological replicates with SD. Two-sided two-way ANOVA, $**p \leqslant 0.01$; $***p \leqslant 0.001$; $****p \leqslant 0.0001$; ns, not significant. Source data are provided as a Source Data file.

Supplementary Figure 6

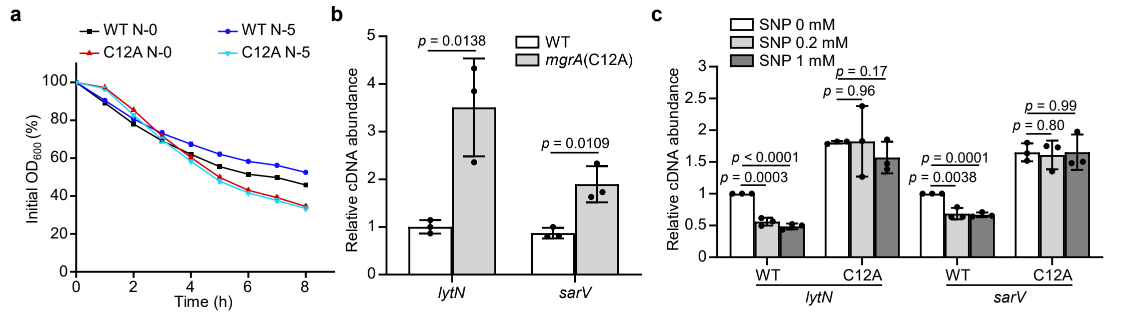

Supplementary Figure 6. The *mgrA*C12A mutant strain showed increased autolysis activity.

a, Triton X-100-induced autolysis of the WT and *mgrA*C12A strains was measured in Tri-HCl buffer supplemented with or without 5 μM SNP (displayed as N-5 or N-0). The percentage of the initial OD_{600} was displayed. Data are means of n = 5 biological replicates with SD. **b**, The transcription levels of *lytN* and *sarV* were tested in the WT and *mgrA*C12A mutant strains by using qRT-PCR. Data are means of n = 3 biological replicates with SD. **c**, The transcription levels of *lytN* and *sarV* were measured in the WT and *mgrA*C12A mutant strains after incubation with different concentrations of SNP as indicated. Data are means of n = 3 biological replicates with SD. Two-sided unpaired Student's t-test, $*p \leqslant 0.05$; $**p \leqslant 0.01$; $***p \leqslant 0.001$; ns, not significant (**b**, **c**). Source data are provided as a Source Data file.

Supplementary Figure 7

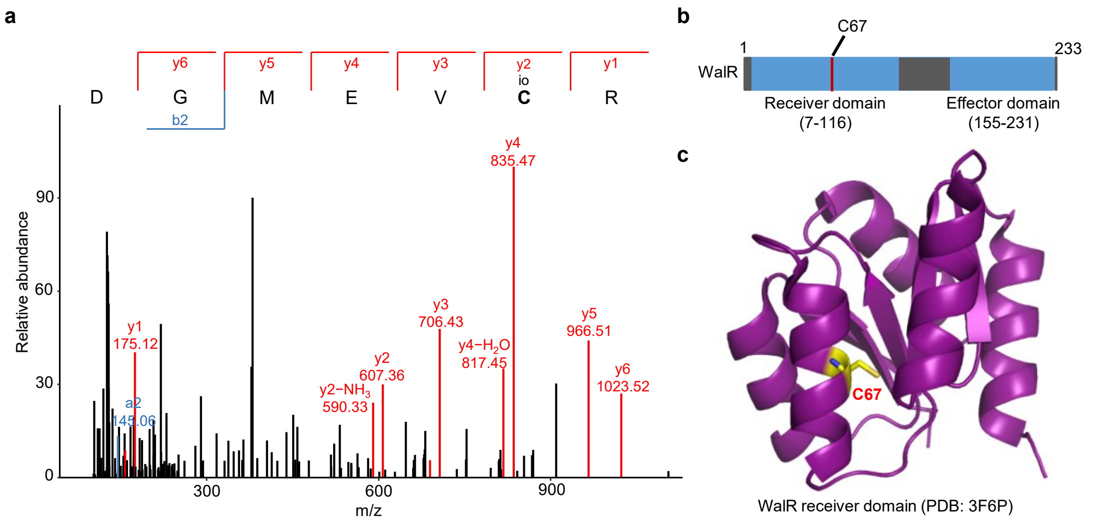

Supplementary Figure 7. Identification of the transcription factor WalR as a target of S-nitrosylation.

a, Higher-energy collision dissociation (HCD) mass spectrum of the cystine 67-containing peptide from WalR. S-nitrosylated cysteine was labeled with isobaric iodoTMT and marked with io. **b-c**, The cysteine residue C67 (yellow) of WalR is located in the receiver domain.

Supplementary Figure 8

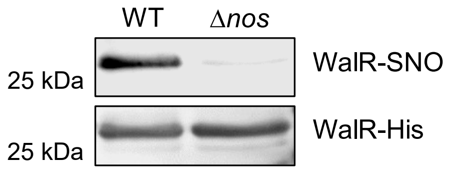

Supplementary Figure 8. Detection of S-nitrosylation of WalR purified from the XN108 WT and Δ*nos* strain by Western blot.

S-nitrosylated WalR (WalR-SNO) was detected with an anti-TMT antibody. Total WalR (WalR-His) was detected with an anti-His antibody. Data are representative of n = 3 biological replicates. Source data are provided as a Source Data file.

Supplementary Figure 9

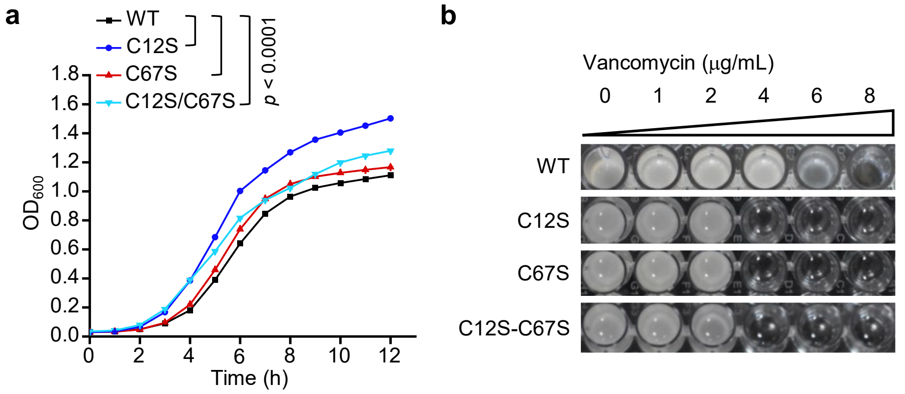

Supplementary Figure 9. The *mgrA*C12S/*walR*C67S mutant strain showed increased vancomycin susceptibility.

a, The growth curve of the WT *S. aureus* XN108, *mgrA*C12S mutant, *walR*C67S mutant and *mgrA*C12S/*walR*C67S mutant strains in TSB medium in 96-well plates with shaking at 200 rpm for 12 h at 37 °C. OD_{600} was monitored to evaluate the growth. Data are means of n = 3 biological replicates with SD. Two-sided two-way ANOVA, $****p \leq 0.0001$. **b,** MICs were assessed after the WT *S. aureus* XN108, *mgrA*C12S mutant, *walR*C67S mutant and *mgrA*C12S/*walR*C67S mutant strains were grown under vancomycin conditions in a 96-well plate with shaking at 200 rpm for 48 h at 37 °C. Data are representative of n = 3 biological replicates. Source data are provided as a Source Data file.

Supplementary Figure 10

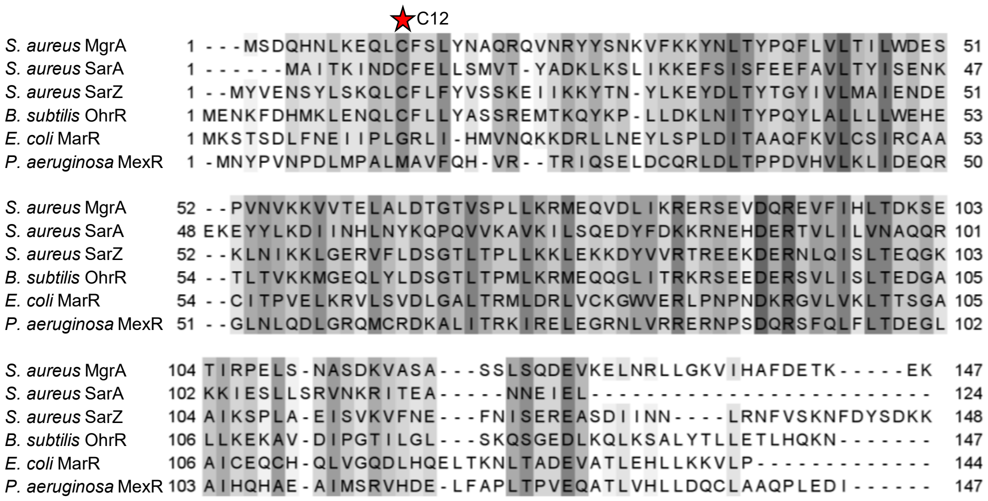

Supplementary Figure 10. Alignment of MgrA sequences with other MarR-type transcriptional regulators.

Multiple sequence alignment of different MarR-type transcriptional regulators was shown as indicated. The alignment was performed using Clustal Omega. Amino acid similarities and identities are shaded gray; the red star shows the position of cysteine residues present in *S. aureus* MgrA in this study.

Full scans of blots in Supplementary Figure 8

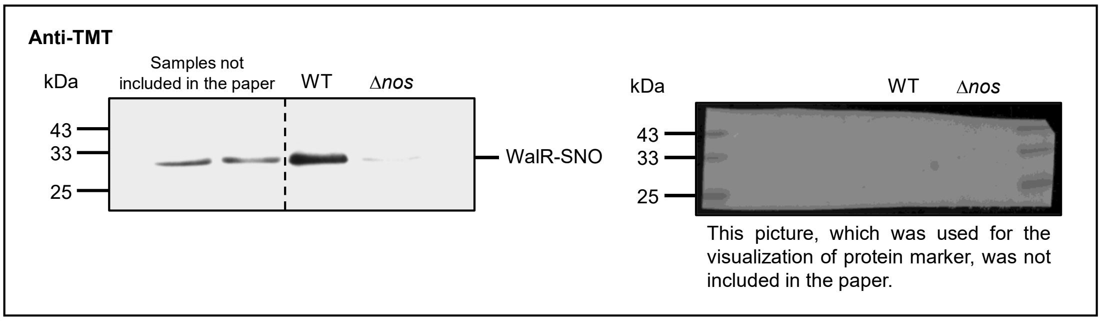

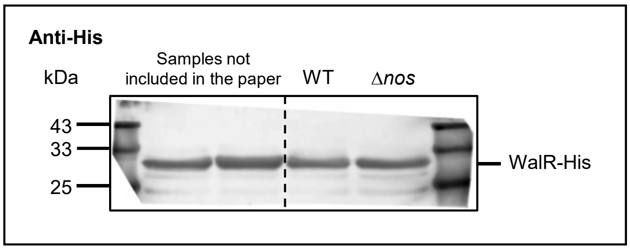

Supplementary Data 1. Original and categorized data of MS identified NO-modified proteins.

Supplementary Table 1. Strains or plasmids used in this study

**Strain or plasmid**	**Description**[a]	**Reference or source**
***S. aureus* strains**		
RN4220	8325-4 r$^-$, initial recipient for modification of plasmids which are introduced into *S. aureus* from *E. coli*	NARSA[b]
XN108	VISA strain, ST239-MRSA	A kind gift from Professor X. Rao
Mu50	VISA strain, ST5-MRSA	NARSA
Δ*nos*	XN108 strain with deletion of *nos*	This study
*mgrA*C12S	XN108 strain with mutation of *mgrA*C12S	This study
*walR*C67S	XN108 strain with mutation of *walR*C67S	This study
*mgrA*C12A	XN108 strain with mutation of *mgrA*C12A	This study
Δ*nos*-*mgrA*C12A	XN108 strain with deletion of *nos* and mutation of *mgrA*C12A	This study
*mgrA*C12S/*walR*C67S	XN108 strain with mutation of *mgrA*C12S and *walR*C67S	This study
***E. coli* strains**		
Trans1-T1	Clone host strain, F$^-$ φ80(*lacZ*) ΔM15Δ*lac*X74 *hsdR*(r_K^-, m_K^+) Δ*recA*1398 *endA*1 *tonA*	TransGen
BL21 (DE3)	Express strain, F$^-$ *ompT hsdSB* (r_B^- m_B^-) gal dcm (DE3)	TransGen
**Plasmids**		
pBTs	Shuttle vector, temp sensitive, Amp^r Cm^r	Lab collection
pET28a(+)	Expression vector with a hexahistidine tag, Kan^r	Novagen
pLI50	Shuttle vector, Amp^r Cm^r	Addgene
pETMgrA	pET28a (+) derivative, with ORF of *mgrA* from strain XN108, Kan^r	This study
pETMgrAC12S	pET28a (+) derivative, with ORF of *mgrA* from strain C12S, Kan^r	This study
pBTs-*nos*	pBTs derivative, for *nos* mutation in strain XN108, Amp^r Cm^r	This study
pBTs-*mgrA*C12S	pBTs derivative, for *mgrA*C12S mutation in strain XN108, Amp^r Cm^r	This study
pBTs-*walR*C67S	pBTs derivative, for *walR*C67S mutation in strain XN108, Amp^r Cm^r	This study
pLIMgrA-His	pLI50 derivative, expression vector of protein MgrA with a hexahistidine tag, Amp^r Cm^r	This study

Strain or plasmid	Description[a]	Reference or source
**Plasmids**		
pLIWalR-His	pLI50 derivative, expression vector of protein WalR with a hexahistidine tag, Amp^r Cm^r	This study

[a] r^-, restriction system negative; Amp^r, ampicillin resistant; Cm^r, chloramphenicol resistant; Kan^r, kanamycin resistant.

[b] NARSA, Network on Antimicrobial Resistance in Staphylococcus aureus.

Supplementary Table 2. Primers used in this study

Primer	Oligonucleotide (5′-3′)	Application
nos-up-F-KpnI	CGGGGTACCGGAATGTATCTACTAGGAACAC	*nos* knockout
nos-up-R	TAACAACACCTCGCTTTATA	*nos* knockout
nos-dn-F	TATAAAGCGAGGTGTTGTTATAAGTTAGTAG AGGTGTAGC	*nos* knockout
nos-dn-R-SacI	CGGGAGCTCCGAGACTCAATCCAGGAT	*nos* knockout
mgrA(C12S)-up-F-KpnI	GGGGTACCGCTTCTATGATGACATGA	*mgrA* point mutation
mgrA(C12S)-up-R	AACTAAAGGATAGCTGTT	*mgrA* point mutation
mgrA(C12S)-dn-F	ACAGCTATCCTTTAGTTT	*mgrA* point mutation
mgrA(C12S)-dn-R-SalI	ACGCGTCGACACTATCAACAATTACTAGC	*mgrA* point mutation
walR(C67S)-up-F-EcoRI	GGAATTCAATTAGATTACCATGTGC	*walR* point mutation
walR(C67S)-up-R	TCACGAGATACTTCCATA	*walR* point mutation
walR(C67S)-dn-F	TGGAAGTATCTCGTGAAG	*walR* point mutation
walR(C67S)-dn-R-KpnI	GGGGTACCGAAGGGATTGTAGTTGTT	*walR* point mutation
MgrA-ex-F-BamHI	CGGGATCCATGTCTGATCAACATAAT	Expression of MgrA
MgrA-ex-R-SalI	ACGCGTCGACATTGTCATGACAAAAGTT	Expression of MgrA
ex-pLI-MgrA-His-F-EcoRI	CGGAATTCCTAATATAATGTGTCAGT	Expression of MgrA
ex-pLI-MgrA-His-R-BamHI	CGGGATCCTTAGTGGTGATGATGGTGATGTT TTTCCTTTGTTTCATC	Expression of MgrA
ex-pLI-WalR-His-F-EcoRI	CGGAATTCACACCAATAGGAATCCCG	Expression of WalR
ex-pLI-WalR-His-R-BamHI	CGGGATCCCTAGTGGTGATGATGGTGATGCT CATGTTGTTGGAGGAA	Expression of WalR
RT-*lytN*-F	TCAGCAATTTCAAACGG	qRT-PCR
RT-*lytN*-R	TGAGCTTCCAAGTTTGC	qRT-PCR
RT-*sarV*-R	TTGTCTTTCATCCGTTTCAG	qRT-PCR
RT-*sarV*-F	CGGTAAAGAATTGAGGGATAC	qRT-PCR
RT-*sle1*-F	CACCAGTATTCAGTCACCAA	qRT-PCR
RT-*sle1*-R	CAGTTATTAGCATTCCACCAAT	qRT-PCR
RT-*atlA*-F	AGCACCAACGGATTAC	qRT-PCR
RT-*atlA*-R	CATACTCAGCACTGTCT	qRT-PCR
RT-*pta*-F	AAAGCGCCAGGTGCTAAATTAC	qRT-PCR
RT-*pta*-R	CTGGACCAACTGCATCATATCC	qRT-PCR
RT-P*lytN*-F	GTAAGGAGTGTTTCATCATG	ChIP-qRT-PCR
RT-P*lytN*-R	GTTCGACATACCCAATGC	ChIP-qRT-PCR
RT-P*sarV*-F	CATTGATGTCACACTCCAT	ChIP-qRT-PCR
RT-P*sarV*-R	GCTGGCTATATCTTGTTTAG	ChIP-qRT-PCR
RT-P*atlA*-F	TCACGTCACCATTGAGATT	ChIP-qRT-PCR
RT-P*atlA*-R	ACCTGTGCGTATTTAACCA	ChIP-qRT-PCR
RT-P*sle1*-F	GTTAGGAAAGTTAAGCAAGAGG	ChIP-qRT-PCR

Primer	Oligonucleotide (5′-3′)	Application
RT-P*sle1*-R	GTGAGTTGTAGCCGCATT	ChIP-qRT-PCR
P*sarV*-F	GTCACACTCCATGCCATT	EMSA
P*sarV*-R-FAM	TGATGCCTCCTATGTTTCTT	EMSA
P*sarV*-R	TGATGCCTCCTATGTTTCTT	EMSA
P*lytN*-F	AAGTATCGGAAATGGGCTAT	EMSA
P*lytN*-R-FAM	GTTCGACATACCCAATGC	EMSA
P*lytN*-R	GTTCGACATACCCAATGC	EMSA
