# Supplementary material for: Transcription tuned by S-nitrosylation underlies a mechanism for Staphylococcus aureus to circumvent vancomycin killing
Source: Nat Commun. 2023 Apr 21;14:2318. doi: 10.1038/s41467-023-37949-0 (PMC10120478; doi:10.1038/s41467-023-37949-0)
Supplement: Supplementary file 3 — Description of Additional Supplementary Files [file 41467_2023_37949_MOESM3_ESM.pdf]

Description of Additional Supplementary Files

File Name: Supplementary Data 1
Description: Original and categorized data of MS identified NO-modified proteins.
