# Supplementary material for: Transcription tuned by S-nitrosylation underlies a mechanism for Staphylococcus aureus to circumvent vancomycin killing
Source: Nat Commun. 2023 Apr 21;14:2318. doi: 10.1038/s41467-023-37949-0 (PMC10120478; doi:10.1038/s41467-023-37949-0)
Supplement: Supplementary file 5 — Reporting Summary [file 41467_2023_37949_MOESM5_ESM.pdf]

nature portfolio

Corresponding author(s): Baolin Sun
Last updated by author(s): Mar 27, 2023

Reporting Summary

Nature Portfolio wishes to improve the reproducibility of the work that we publish. This form provides structure for consistency and transparency in reporting. For further information on Nature Portfolio policies, see our Editorial Policies and the Editorial Policy Checklist.

Statistics

For all statistical analyses, confirm that the following items are present in the figure legend, table legend, main text, or Methods section.

n/a	Confirmed	
☐	☒	The exact sample size (*n*) for each experimental group/condition, given as a discrete number and unit of measurement
☐	☒	A statement on whether measurements were taken from distinct samples or whether the same sample was measured repeatedly
☐	☒	The statistical test(s) used AND whether they are one- or two-sided<br>*Only common tests should be described solely by name; describe more complex techniques in the Methods section.*
☒	☐	A description of all covariates tested
☒	☐	A description of any assumptions or corrections, such as tests of normality and adjustment for multiple comparisons
☐	☒	A full description of the statistical parameters including central tendency (e.g. means) or other basic estimates (e.g. regression coefficient) AND variation (e.g. standard deviation) or associated estimates of uncertainty (e.g. confidence intervals)
☐	☒	For null hypothesis testing, the test statistic (e.g. *F*, *t*, *r*) with confidence intervals, effect sizes, degrees of freedom and *P* value noted<br>*Give P values as exact values whenever suitable.*
☒	☐	For Bayesian analysis, information on the choice of priors and Markov chain Monte Carlo settings
☒	☐	For hierarchical and complex designs, identification of the appropriate level for tests and full reporting of outcomes
☒	☐	Estimates of effect sizes (e.g. Cohen's *d*, Pearson's *r*), indicating how they were calculated

Our web collection on statistics for biologists contains articles on many of the points above.

Software and code

Policy information about availability of computer code

Data collection	Excel (Microsoft 2021), Origin (2021b)
Data analysis	Excel (Microsoft 2021), Prism 9 (GraphPad)

For manuscripts utilizing custom algorithms or software that are central to the research but not yet described in published literature, software must be made available to editors and reviewers. We strongly encourage code deposition in a community repository (e.g. GitHub). See the Nature Portfolio guidelines for submitting code & software for further information.

Data

Policy information about availability of data

All manuscripts must include a data availability statement. This statement should provide the following information, where applicable:

- Accession codes, unique identifiers, or web links for publicly available datasets
- A description of any restrictions on data availability
- For clinical datasets or third party data, please ensure that the statement adheres to our policy

A reporting summary for this article is available as Supplementary Information file. The main data supporting the findings of this study are available within the article and its supplementary information files. Additional details on datasets and protocols that support the findings of this study will be made available by the corresponding author upon reasonable request. The raw data of the proteomics generated in this study have been deposited in iProX database under ProjectID IPX0006165000 and are publicly available. Source data are provided with this paper. These information has also been provided in the manuscript under the “Data availability” section.

Field-specific reporting

Please select the one below that is the best fit for your research. If you are not sure, read the appropriate sections before making your selection.

☒ Life sciences ☐ Behavioural & social sciences ☐ Ecological, evolutionary & environmental sciences

For a reference copy of the document with all sections, see nature.com/documents/nr-reporting-summary-flat.pdf

Life sciences study design

All studies must disclose on these points even when the disclosure is negative.

Sample size	Sample sizes were determined according to current standards used for all the experiments in this study based on the minimal amount of samples required to detect significance with an alpha set at 0.05 in an experiment.
Data exclusions	N/A
Replication	Biological replicates are described in the figure legends.
Randomization	The clones of strains picked for experiments were randomized based on clone size and shape.
Blinding	Experimenters were blinded to group allocations during experiments/outcome assessment.

Reporting for specific materials, systems and methods

We require information from authors about some types of materials, experimental systems and methods used in many studies. Here, indicate whether each material, system or method listed is relevant to your study. If you are not sure if a list item applies to your research, read the appropriate section before selecting a response.

Materials & experimental systems

n/a	Involved in the study	
☐	☒	Antibodies
☒	☐	Eukaryotic cell lines
☒	☐	Palaeontology and archaeology
☒	☐	Animals and other organisms
☒	☐	Human research participants
☒	☐	Clinical data
☒	☐	Dual use research of concern

Methods

n/a	Involved in the study	
☒	☐	ChIP-seq
☒	☐	Flow cytometry
☒	☐	MRI-based neuroimaging

Antibodies

Antibodies used

Anti-His antibody (BOSTER, Cat#M30975, Clone Number 9C11, 1:1000);
Anti-TMT antibody (Pierce, Cat#90075, Clone Number 25D5, 1:1000);
Anti-MgrA antibody (Specifically prepared commercially by MerryBio with no catalog number);
Anti-WalR antibody (Specifically prepared commercially by Genscript with no catalog number);
Normal Rabbit IgG (Cell Signaling Technology, Cat#2729S).

Validation

Western-blot
Anti-His antibody (BOSTER, Cat#M30975, 1:1000)
Species: Mouse
Applications: Dot blot, ELISA, WB

Western-blot
Anti-TMT antibody (Pierce, Cat#90075, 1:1000)
Species: Mouse / IgG2b
Applications: WB, IP

IImmunoprecipitation
Anti-MgrA antibody (MerryBio, This antibody was specifically generated by the company for our experiments thus has no catalog number.)
Species: Rabbit
Applications: We have validated this antibody by performing WB and IP experiments using this antibody.

Immunoprecipitation
Anti-WalR antibody (Genscript, This antibody was specifically generated by the company for our experiments thus has no catalog

number.)
Species: Rabbit
Applications: We have validated this antibody by performing WB and IP experiments using this antibody.

Normal Rabbit IgG (Cell Signaling Technology, Cat#2729S)
Species: Rabbit
Applications: IP, ChIP
